# Inhibition of colony stimulating factor-1 receptor improves antitumor efficacy of BRAF inhibition

**DOI:** 10.1186/s12885-015-1377-8

**Published:** 2015-05-05

**Authors:** Stephen Mok, Jennifer Tsoi, Richard C Koya, Siwen Hu-Lieskovan, Brian L West, Gideon Bollag, Thomas G Graeber, Antoni Ribas

**Affiliations:** 1 Department of Molecular and Medical Pharmacology, University of California Los Angeles (UCLA), Los Angeles, CA USA; 2 Department of Surgery, Division of Surgical Oncology, University of California Los Angeles (UCLA), Los Angeles, CA USA; 3 Department of Medicine, Division of Hematology/Oncology, UCLA, University of California Los Angeles (UCLA), 11-934 Factor Building, 10833 Le Conte Avenue, Los Angeles, CA 90095-1782 USA; 4 Crump Institute for Molecular Imaging, UCLA, University of California Los Angeles (UCLA), Los Angeles, CA USA; 5 Jonsson Comprehensive Cancer Center (JCCC), University of California Los Angeles (UCLA), Los Angeles, CA USA; 6 Plexxikon Inc, Berkeley, California USA; 7 MD Anderson Cancer Center, Houston, Texas USA; 8 Roswell Park Cancer Institute, Buffalo, New York USA

**Keywords:** PL3397, PLX4032, T-cell, Tumor microenvironment, Macrophage

## Abstract

**Background:**

Malignant melanoma is an aggressive tumor type that often develops drug resistance to targeted therapeutics. The production of colony stimulating factor 1 (CSF-1) in tumors recruits myeloid cells such as M2-polarized macrophages and myeloid derived suppressor cells (MDSC), leading to an immune suppressive tumor milieu.

**Methods:**

We used the syngeneic mouse model of *BRAF*^*V600E*^-driven melanoma SM1, which secretes CSF-1, to evaluate the ability of the CSF-1 receptor (CSF-1R) inhibitor PLX3397 to improve the antitumor efficacy of the oncogenic BRAF inhibitor vemurafenib.

**Results:**

Combined BRAF and CSF-1R inhibition resulted in superior antitumor responses compared with either therapy alone. In mice receiving PLX3397 treatment, a dramatic reduction of tumor-infiltrating myeloid cells (TIM) was observed. In this model, we could not detect a direct effect of TIMs or pro-survival cytokines produced by TIMs that could confer resistance to PLX4032 (vemurafenib). However, the macrophage inhibitory effects of PLX3397 treatment in combination with the paradoxical activation of wild type *BRAF*-expressing immune cells mediated by PLX4032 resulted in more tumor-infiltrating lymphocytes (TIL). Depletion of CD8+ T-cells abrogated the antitumor response to the combination therapy. Furthermore, TILs isolated from SM1 tumors treated with PLX3397 and PLX4032 displayed higher immune potentiating activity.

**Conclusions:**

The combination of BRAF-targeted therapy with CSF-1R blockade resulted in increased CD8 T-cell responses in the SM1 melanoma model, supporting the ongoing evaluation of this therapeutic combination in patients with *BRAF*^*V600*^ mutant metastatic melanoma.

**Electronic supplementary material:**

The online version of this article (doi:10.1186/s12885-015-1377-8) contains supplementary material, which is available to authorized users.

## Background

Targeted therapies such as the oncogenic BRAF inhibitor PLX4032 (with generic drug name vemurafenib) has resulted in high response rates and improved overall survival in patients with melanoma. However, consistent with other oncogene-targeted therapies, initial patient response is of limited durability and tumors eventually relapse [1-4].

Overcoming the immunosuppressive tumor microenvironment mediated by growth factor and receptor tyrosine kinases (RTK) have been of particular interest in cancer therapy. Tumor cells manipulate the surrounding milieu by producing cytokines that suppress cytolytic T-cells and recruit immunosuppressive cells [5-7]. Colony stimulating factor 1 (CSF-1) is one such cytokine secreted by several cancer cell types, including melanoma [8,9]. It induces the proliferation and differentiation of immunosuppressive myeloid cells such as M2 polarized macrophages and myeloid derived suppressor cells (MDSC) by binding to the CSF-1 receptor (CSF-1R) on the cell surface [5,10,11]. Therefore, the immunosuppressive tumor milieu mediated by CSF-1 helps tumor cells escape immune responses and metastasize.

In prior studies, we developed the SM1 cell line from transgenic mice with melanocyte-restricted expression of the *BRAF*^*V600E*^ mutation. SM1 is a murine melanoma model syngeneic to immunocompetent mice. In addition to carrying the *BRAF*^*V600E*^ oncogene, SM1 has multiple genomic aberrations and share overall similarity to a panel of 108 human melanoma cell lines based on copy number alteration profiling. In this model, we observed that adoptive cell transfer (ACT) of melanoma-targeted T-cells induces antitumor responses that are augmented by the BRAF inhibitor PLX4032. Treatment with PLX4032 induces paradoxical activation on TILs, resulting in increased cytotoxic activity and IFN-γ production [12]. These findings provide a potential explanation as to why host immunity is a key component of the antitumor activity of BRAF inhibitors [13]. However, the SM1 model is an aggressive model in which control mice with established tumors need to be sacrificed within two to three weeks. Accordingly, tumors could not be fully eradicated when immunotherapy was combined with PLX4032. In addition, our previous work demonstrated that SM1 cells secrete cytokines such as CSF-1 that binds to CSF-1R on myeloid cells to recruit and promote the differentiation of myeloid cells into immunosuppressive M2-polarized macrophages. On the therapeutic front, we report that PLX3397, a potent tyrosine kinase inhibitor that targets CSF-1R, inhibits the immunosuppressive tumor milieu and facilitates immune responses, resulting in improved antitumor T-cell function [14].

In this report, we demonstrate that the combination of PLX4032 and PLX3397 mediates superior antitumor responses compared with either single treatment alone. PLX3397 treatment blocked the recruitment of TIMs and increased the number of TILs. We observed that full antitumor efficacy of PLX4032 required an intact immune system. Taken together, our data support a model in which inhibition of CSF-1/CSF-1R signaling can augment the antitumor effect of BRAF targeted therapy. Based on our results in the SM1 model, we provide preclinical support for the therapeutic combination of BRAF and CSF-1R inhibition currently being tested in patients with *BRAF*^*V600*^ mutant metastatic melanoma (trial NCT01826448).

## Methods

### Mice, cell lines and reagents

C57BL/6 mice and NOD/SCID/γ chain^null^ (NSG) mice (NOD.Cg-*Prkdc*^scid^*Il2rg*^tm1Wjl^/SzJ, Jackson Laboratory, Bar Harbor, ME) were bred and kept under defined-flora pathogen-free conditions at the AALAC-approved animal facility of the Division of Experimental Radiation Oncology, UCLA, and used under the UCLA Animal Research Committee protocol #2004-159. The SM1 murine melanoma was generated from a spontaneously arising tumor in *BRAF*^*V600E*^ mutant transgenic mice as previously described [15]. SM1 was maintained in RPMI (Mediatech, Herndon, VA) with 10% FCS (Omega Scientific), 2 mM L-glutamine (Invitrogen, Carlsbad, CA) and 1% penicillin, streptomycin and amphotericin. Immortalized macrophages I-11.15 were obtained from ATCC and were maintained as previously described [16]. PLX3397 and PLX4032 were obtained under a materials transfer agreement (MTA) with Plexxikon Inc. (Berkeley, CA). PLX3397 was dissolved in dimethyl sulfoxide (DMSO, Fisher Scientific, Morristown, NJ) for *in vitro* use. For *in vivo* studies, PLX3397 was dissolved in DMSO, and then a suspension made by dilution into an aqueous mixture of 0.5% hydroxypropyl methyl cellulose (HPMC) and 1% polysorbate (PS80) (Sigma-Aldrich). 100 μL of the suspended drug was administered by daily oral gavage into mice at 50 mg/kg when tumors reached 5 mm in diameter. PLX4032 was dissolved in DMSO, and used for in vitro studies as previously described [17]. For *in vivo* studies, it was dissolved in DMSO, followed by PBS (100 μL), which was then injected daily intraperitoneally (i.p) into mice at a dose of 100 mg/kg. For antibody-mediated depletion studies, 250 μg of anti-CD8 antibody, or isotype control antibody (BioXCell, West Lebanon, NH) was injected i.p. every 3 days.

### Cell viability assays

SM1 cells (5 × 10^3^ cells/well) were seeded on 96-well flat-bottom plates with 100 μL of 10% FCS media and incubated for 24 hours. PLX4032 or DMSO vehicle control with graded dilutions of hepatocyte growth factor (HGF) or tumor necrosis factor-α (TNF-α) (PeproTech), in culture medium, were added to each well in triplicate and analyzed by using tetrazolium compound [3-(4,5-dimethylthiazol-2-yl)-5-(3-carboxymethoxyphenyl)-2-(4-sulfophenyl)-2H-tetrazolium (MTS)-based colorimetric cell proliferation assay (Promega, Madison, WI).

### Bioluminescence assay

SM1 cells were lentivirally transduced to express firefly luciferase and used for co-culturing with macrophages. Bioluminescence assays were carried out with a DTX880 Multimode Detector (Beckman Coulter).

### Flow cytometry analysis and cell sorting

SM1 tumors were harvested from mice and further digested with collagenase (Sigma-Aldrich). Cells obtained form digested SM1 tumors were stained with antibodies to CD3, CD8 (BD Biosciences) for TILs or antibodies to F4/80, CD11b for TIMs and analyzed with a LSR-II or FACSCalibur flow cytometer (BD Biosciences), followed by Flow-Jo software (Tree-Star, Ashland, OR) analysis as previously described [12,14].

### Immunofluorescence imaging

Staining was performed as previously described [15]. Briefly, sections of OCT (Sakura Finetek, Torrance, CA) cryopreserved tissues were blocked in donkey serum/ PBS and incubated with primary antibodies to F4/80 (Abcam) or CD8 (BD Biosciences), followed by secondary donkey anti-rat antibodies conjugated to DyLight488 (Jackson Immunoresearch Laboratories, West Grove, PA). Negative controls consisted of isotype matched rabbit or rat IgG in lieu of the primary antibodies listed above. DAPI (4,6-diamidino-2-phenylindole) was used for the visualization of nuclei. Immunofluorescence images were taken in a fluorescence microscope (Axioplan-2; Carl Zeiss Microimaging, Thornwood, NY).

### Intratumoral myeloid cell isolation

SM1 tumors previously established in C57BL/6 mice were harvested and further digested with collagenase (R&D System). Intratumoral myeloid cells were isolated from digested tumor using CD11b + cell isolation kit (Miltenyi).

### Microarray data generation and analysis

Total RNAs were extracted using the RNeasy MicroKit (Qiagen) from SM1 tumors, FACS-sorted macrophages, and T-cells. Yield of RNA was amplified using RNA Amplification System (NuGEN). cDNAs were generated, fragmented, biotinylated, and hybridized to the GeneChip Mouse 430 V2 Arrays (Affymetrix). The arrays were washed and stained on a GeneChip Fluidics Station 450 (Affymetrix); scanning was carried out with the GeneChip Scanner 3000 7G; and image analysis with the Affymetrix GeneChip Command Console Scan Control. Microarray analyses were performed in the R statistical programming environment and using Bioconductor suite of packages [18]. Expression data were normalized, background-corrected, and summarized using the Robust Multi-Array Average (RMA) algorithm implemented in the R ‘affy’ package [19]. Two to three replicates were prepared per treatment group. Expression level of each gene was averaged among samples and used for further analysis. Differential expression was computed using the R ‘limma’ package [20]. Hierarchical clustering was performed using the Euclidean distance as the similarity metric with average linkage clustering. Clustering results were visualized by heat maps generated using the R ‘NMF’ package [21].

### Rank-rank hypergeometric overlap (RRHO) analysis

Gene expression profiles of monocytes and T-cells were obtained from a reference immune cell signature database, the Differentiation Map Portal (DMAP) [22]. Human gene annotations were converted to mouse gene annotations using the NCBI HomoloGene database. Gene-expression profiles from the two data sets were compared by ranking genes measured in the two experiments according to their signed log10 p-value of differential expression between class A and class B. RRHO heat maps that graphically and statistically visualize correlations between two expression profiles were generated at http://systems.crump.ucla.edu/rankrank/ [23].

### Gene ontology enrichment analysis

Gene ontology enrichment analysis was performed on genes with a log_2_ fold change greater than 2.0 in the combo treated groups using the GOTermFinder tool from http://go.princeton.edu/cgi-bin/GOTermFinder [24]. Top significantly enriched terms as determined by Bonferroni corrected p-values were reported.

### Statistical analysis

Data were analyzed with GraphPad Prism (version 5) software (GraphPad Software, La Jolla, CA). A Mann–Whitney test or ANOVA with Bonferroni post-test was used to analyze experimental data. Survival curves were generated by actuarial Kaplan–Meier method and analyzed with the Jump-In software (SAS) with log-rank test for comparisons from the time of tumor challenge to when mice were sacrificed due to tumors reaching 14 mm in maximum diameter, or when the end of the study period had been reached.

## Results

### Combined therapy with PLX3397 and PLX4032 improves antitumor responses against SM1 tumors

**Figure 1:**
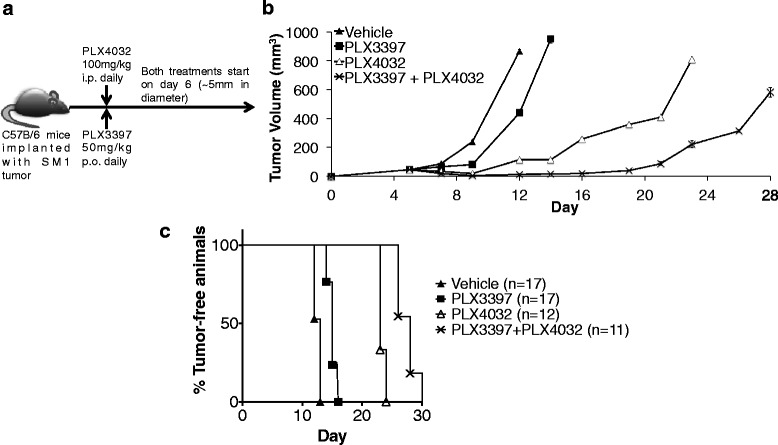
Combined antitumor activity of PLX3397 and PLX4032 in murine melanoma SM1 model. **a)** Schematic of PLX3397 and PLX4032 treatments in C57BL/6 mice with previously established SM1 tumors. **b)** Tumor growth curves of established SM1 in C57BL/6 mice through day 28 post-tumor implantation. On day 12, the differences between vehicle and PLX3397: p = 0.0000004; PLX3397 and PLX4032: p = 0.000000009; PLX4032 and combo: p = 0.000001. **c)** Kaplan–Meier actuarial plot of time to mouse sacrifice due to large tumor burden or to study termination when tumor size was less than 14 mm in maximum diameter.

C57BL/6 mice with established subcutaneous SM1 tumors were treated with the CSF-1R inhibitor PLX3397 and the BRAF inhibitor PLX4032 daily once tumor diameter reached ~5 mm (Figure 1a). The combined therapy of PLX3397 and PLX4032 demonstrated superior antitumor effects compared to either therapy alone in duplicate experiments and improved overall survival (Figure 1b and c). Mice in either of the single or combined treatment groups had high drug tolerability as determined by no significant weight loss (data not shown).

### Decrease in tumor infiltrating macrophages by PLX3397 and PLX4032

**Figure 2:**
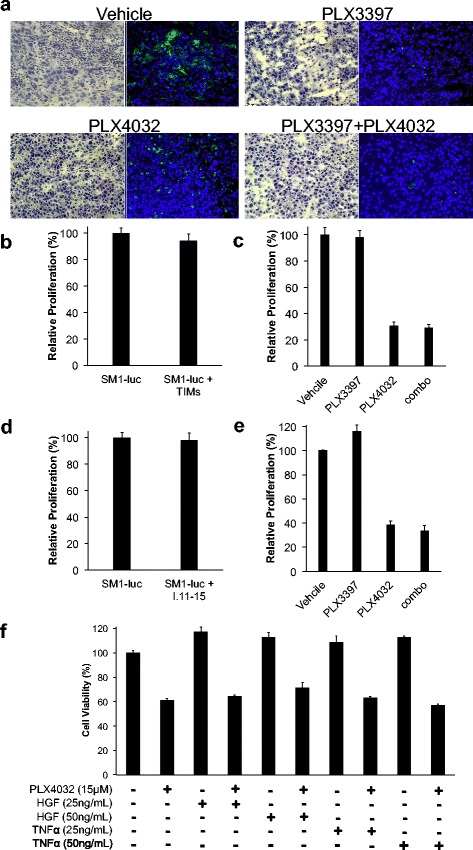
Changes in intratumoral macrophages in responses to PLX3397 and PLX4032. **a)** C57BL/6 mice with SM1 tumors were treated with PLX3397 and PLX4032 for 5 days. Tissue immunofluorescence microscopy of tumor sections was used to assess prolonged effects of the drug on macrophages. Representative H&E (left) and immunofluorescence for macrophages stained with anti-F4/80-FITC (green, right), and nuclei stained with DAPI (blue, right). **b)** Effect of macrophages on SM1 cells. Bar-graph representation of bioluminescence activity of SM1 cells. SM1 cells were transduced with a lentivirus-firefly luciferase and co-cultured with and without intratuoral myeloid cells isolated from SM1 tumors (1:3 ratio) for 72 hours. Difference between SM1-luc and SM1-luc + TIMs: p = 0.4 **c)** SM1 cells co-cultured with myeloid cells (1:3 ratio) were treated with 1 μM PLX3397, 15 μM PLX4032, or in combination for 72 hours. Difference between vehicle and PLX3397: p = 0.83; PLX3397 and PLX4032: p = 0.0001; PLX4032 and combo: p = 0.39. **d)** SM1 cells expressing firefly luciferase were co-cultured with I-11.15 (1:3 ratio). Difference between SM1-luc and SM1-luc + I-11.15: p = 0.6. **e)** SM1-luc cells co-cultured with I-11.15 were treated with 1 μM PLX3397, 15 μM PLX4032, or in combination for 72 hours. Difference between vehicle and PLX3397: p = 0.05; PLX3397 and PLX4032: p = 0.0002; PLX4032 and combo: p = 0.7. **f)** Effect of growth factors on SM1 to PLX4032. SM1 cells were exposed to 15 μM PLX4032 with HGF or TNF-α (25 or 50 ng/mL). Cell viability assay (MTS) was performed after 72 hours. Difference between vehicle and PLX4032: p = 0.004; PLX4032 and PLX4032 + HGF (50 ng/mL): p = 0.06; PLX4032 and PLX4032 + TNF-α (50 ng/mL): p = 0.12.

To test whether PLX3397 and PLX4032 changed the number of TIMs such as macrophages, we analyzed their presence in tumors by immunofluorescence. The results corresponded to our previous findings of a dramatic decrease in the quantity of F4/80(+) macrophages in both the PLX3397 single agent group and the combined group compared to vehicle control [14]. PLX4032 slightly decreased the number of macrophages in the tumor (Figure 2a), in agreement with previous reports [13,25,26].

### Effects of macrophages on SM1 cells

It has been reported that some cell types in the tumor microenvironment such as stromal cells can secrete growth factors like hepatocyte growth factor (HGF) or tumor necrosis factor-α (TNF-α) resulting in resistance to BRAF inhibition [27-29]. A potential mechanism to explain the improved antitumor activity of combining PLX3397 with PLX4032 is that PLX3397 depletes macrophages, which secrete pro-survival growth factors, and thus increases the sensitivity of SM1 tumor cells to PLX4032. To test this hypothesis, SM1 cells were transduced to express firefly luciferase and co-cultured with intratumoral myeloid cells obtained from mice. We tested whether the presence of TIMs could increase proliferation of SM1 by producing secreted factors that would foster melanoma cell growth. However, we found that the co-cultured myeloid cells did not directly increase SM1 proliferation (Figure 2b). Furthermore, in this context PLX3397 did not increase sensitivity of SM1 cells to PLX4032 (Figure 2c). We next repeated this experiment using an immortalized macrophage cell line, I-11.15, in which macrophage cell growth is dependent on secreted CSF-1. Again, co-culture with I-11.15 cells did not increase proliferation of SM1 nor protect it from PLX4032 (Figure 2d and e). In order to further test if secreted growth factors mediate resistance to BRAF inhibition, SM1 cells were cultured with HGF or TNF-α and treated with PLX4032. Using a MTS-based assay to determine cell viability, we found that neither of these cytokines mediated resistance to PLX4032 (Figure 2f). From these studies, we concluded that the direct effect of TIMs or pro-survival cytokines produced by TIMs did not confer resistance to PLX4032.

### PLX3397 increases the expansion of intratumoral lymphocytes

**Figure 3:**
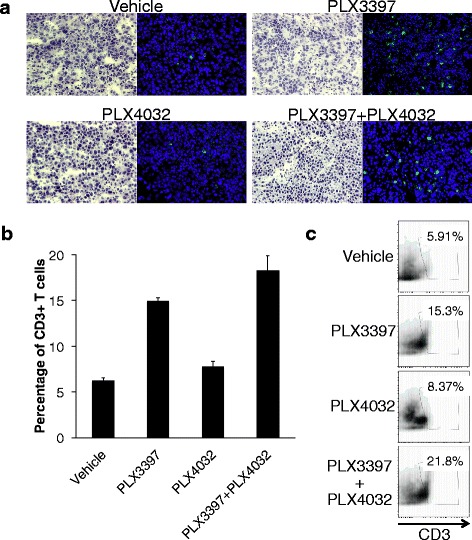
Changes in tumor infiltrating lymphocytes in responses to PLX3397 and PLX4032. **a)** C57BL/6 mice with SM1 tumors were treated with PLX3397 and PLX4032 for 5 days. Tissue immunofluorescence microscopy of tumor sections was used to determine effect of the drugs on lymphocytes. Representative H&E (left) and immunofluorescence for lymphocytes stained with anti-CD8-FITC (green, right), and nuclei stained with DAPI (blue, right). **b)** Cells stained for CD3 were used for FACS analysis. Bar-graph representation of percentage of CD3+ T-cells in tumors. **c)** Representative FACS plots demonstrating percentages of CD3+ T-cells in tumor tissue.

The amount of TILs in SM1 tumors was first analyzed by immunofluorescence of tumor sections. CD8(+) TILs were present at a low level in tumors from vehicle and PLX4032 single treatment groups and at high levels in the PLX3397 and combined treatment groups (Figure 3a). To better enumerate the magnitude and distribution of TILs *in vivo*, we analyzed their presence in tumors by flow cytometry. Consistent with the immunofluorescence data, there was an increase in the quantity of CD3(+) TILs following treatment with PLX3397 (Figure 3b and c).

### The antitumor activity of PLX3397 + PLX4032 is T-cell-dependent

**Figure 4:**
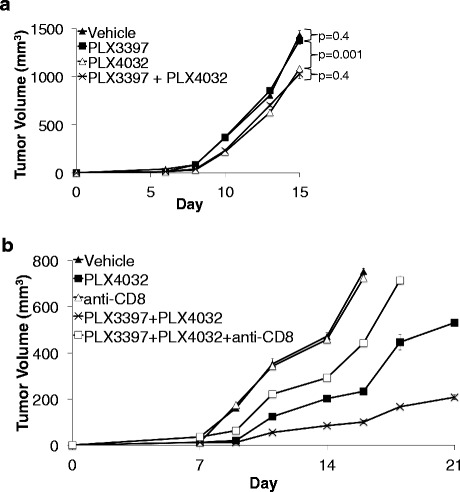
Lack of superior antitumor activity of PLX3397 and PLX4032 in immunodeficient mice or with CD8+ T-cell depletion. **a)** Tumor growth curves of established SM1 tumors in NSG mice treated with PLX3397 and PLX4032. **b)** SM1 tumor-bearing mice treated with PLX3397 and PLX4032 received anti-CD8 depleting antibody. On day 16, the differences between vehicle and anti-CD8 depletion group: p = 0.15; anti-CD8 depletion group and triple combination: p = 0.0000001; triple combination and PLX4032: p = 0.0000001; PLX4032 and combo: p = 0.000006.

Since PLX3397 treatment increased the number of TILs compared to vehicle control (Figure 3a, b, and c), we tested the role of immune cells in the antitumor activity of PLX3397 and PLX4032. Immunodeficient NSG mice were implanted with SM1 tumors and treated with PLX3397 and PLX4032. In these immunodeficient mice, there was no antitumor activity of PLX3397 compared to mice receiving vehicle control. Furthermore, the tumor growth curve of the combined treatment group overlapped with the PLX4032 alone group (Figure 4a). Surprisingly, tumor growth of SM1 tumors in the PLX4032 alone group was much faster in NSG mice than C57BL/6 mice, supporting the possibility that the full antitumor effect of PLX4032 requires an intact immune system (Figures 1b and 4a). In order to further determine if endogenous cytotoxic CD8+ T-cells mediated the antitumor activity of the combined treatment group, we depleted CD8+ cells using anti-CD8 antibody therapy in C57BL/6 mice implanted with SM1 tumors and receiving PLX3397 and PLX4032. The depletion of CD8+ cells abrogated the antitumor activity of the combined treatment group (Figure 4b). Collectively, these studies highlight the role of CD8+ T-cells as effectors of the antitumor activity of PLX3397 and PLX4032 in the SM1 murine melanoma model.

### PLX3397 and PLX4032 increase functional activation of intratumoral lymphocytes and suppress myeloid cells

**Figure 5:**
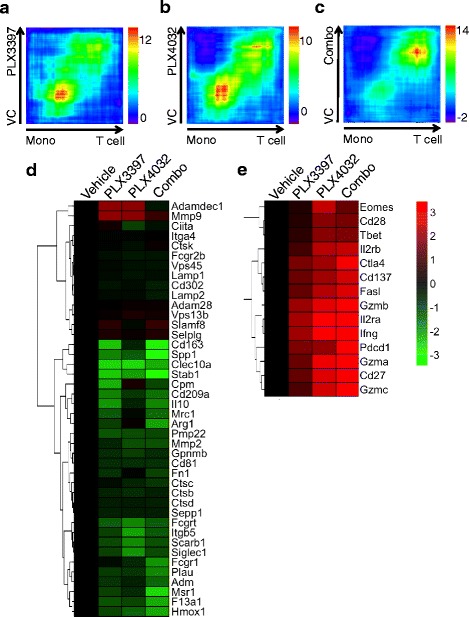
*In vivo* T-cell activation and suppression of macrophages mediated by PLX3397 and PLX4032. Gene expression signature overlap maps were used to compare the similarity of drug-treated full tumor gene expression profiles with a panel of T-cell and monocyte (Mono) gene signatures from a reference immune cell signature database (the Rank-Rank Hypergeometric Overlap (RRHO) algorithm and the Differentiation Map (DMAP) database), **a)** PLX3397 treatment vs vehicle control (VC); **b)** PLX4032 vs vehicle; **c)** combined drug treatment (Combo) vs vehicle. **d)** Gene expression heat map for macrophage signature genes in F4/80(+) CD11b(+) macrophages FACS-sorted from SM1 tumors treated with PLX3397 or PLX4032 for 5 days. **e)** Gene expression heat map for T-cell signature genes in CD3(+) CD8(+) T-cells from SM1 tumors. Color scale, log_2_-transformed fold change expression (red, high; green, low) for each gene (row) normalized to the value for the vehicle control-treated tumors.

In two prior reports [12,14], we have demonstrated that PLX4032 increased cytotoxicity and the cytokine production function of T-cells, while PLX3397 enhanced T-cell cytokine production and infiltration into tumors. In order to better understand the impact of PLX3397 and PLX4032 on T-cell activation and macrophage suppression, we compared the gene expression profile of SM1 tumors following treatments with PLX3397, PLX4032, or combination treatment for 5 days. Using a gene signature overlap analysis (Rank-Rank Hypergeometric Overlap, RRHO) and a reference immune cell signature database (Differentiation Map, DMAP), we analyzed the presence of T-cell and monocytes gene signatures in each tumor treatment group compared with vehicle control [22,23]. The signature comparison analysis identified that the drug-treated tumors expressed T-cell signatures, while the vehicle-treated tumors expressed stronger monocyte signatures (Figure 5a, b, and c). These findings suggest that PLX3397 and PLX4032 combined treatment groups had increased T-cell infiltration and decreased monocyte cell presence or activity in the tumor. Of note, there was no significant difference in this signature analysis between the single drug treatment groups and the combined drug treatment group.

In order to specifically determine the expression level of genes in tumor-associated macrophages and T-cells, F4/80(+) CD11b(+) macrophages and CD3(+) CD8(+) T-cells were FACS sorted from SM1 tumors. Tumors were either treated with vehicle, PLX3397 and/or PLX4032 for 5 days. RNA was extracted and used for microarray gene expression analysis. Since IFN-γ provides a general assessment of T-cell immune activation, the expression values of *IFN-γ* was used as a quality control for this study (Additional file 1: Figure S1c). We found that M2-polarized macrophage-related genes reported previously [30,31], such as *Arg1*, *IL-10*, *CD163*, and *MSR1* were down-regulated in each of the drug-treatment groups (Figure 5d). In contrast, genes, such as *IFN-γ*, *Irg1*, and *Gbp1* that are associated with an type I interferon response were up-regulated in macrophages in both PLX3397 and PLX4032 treatment groups (Additional file 1: Figure S1a; Table S1) [32]. Using gene ontology-based enrichment analysis, we found that the genes most substantially down-regulated in macrophages were associated with angiogenesis and vasculature development (Additional file 1: Figure S1b; Table S2). We also observed that PLX3397, PLX4032 and combined treatment led to an improved T-cell activation signature. For example, T-cell activation-associated genes such as *IFN-γ, Gzmb, Pdcd1* were up-regulated in T-cells in response to all drug treatments (Figure 5e; Additional file 1: Figure S1c) [33,34]. Collectively, our data suggests that PLX3397 and PLX4032 not only down-regulate M2-polarized macrophage associated genes, but also induce a skewing toward M1-type macrophages that results in increased T-cell activation.

## Discussion

The high initial antitumor efficacy of BRAF inhibitors is limited by the short durability of responses. Engaging an immune response may merge the benefits of the high response rates of BRAF inhibitors and the long-term response durability of immunotherapy [35]. Our data support the combination of CSF-1R inhibition with PLX4032 and provide a strong rationale to translate combined targeted therapy and inhibition of myeloid cells for patients with *BRAF*^*V600*^ mutant metastatic melanoma. The scientific rationale for this combination is based on our results from two previous reports [12,14] using a murine melanoma model SM1 that has the *BRAF*^*V600E*^ mutation. Besides causing apoptosis and cell cycle arrest in *BRAF*^*V600E*^ mutant melanoma cells, in BRAF wild type cells PLX4032 has also been shown to have the paradoxical effect of activating the MAPK pathway through the transactivation of CRAF by a partially blocked wild-type CRAF-BRAF dimer. This results in increased T-cell activation with increased cytotoxic activity and intratumoral cytokine secretion from TILs [12,36,37]. At the same time, PLX3397 has been demonstrated to increase the number of TILs with enhanced IFN-γ secreting function [14]. Here we found the combination of both pharmacologic interventions augments the individual antitumor effects.

We explored the potential mechanisms by which PLX3397 improves the antitumor effect of PLX4032 using the SM1 melanoma tumor model. Our studies showed that PLX3397 dramatically depleted macrophages in the tumor microenvironment and PLX4032 slightly decreased the number of macrophages, likely due to down-regulation of secreted cytokines such as CCL2 that could recruit immunosuppressive cells [13]. Furthermore, we noted an increase in the number of TILs in tumors treated with PLX3397. Depletion of CD8+ T-cells in mice abrogated the superior antitumor effect of the combined therapy, providing support for the role of T cells in the observed antitumor effect. Furthermore, TILs from either PLX3397 or PLX4032 treatment groups had higher functional activation with increased ability to release the immune-stimulating cytokine IFN-γ compared to the untreated group. The immune-activating effects mediated by PLX4032 can be explained by the paradoxical activation of MAPK pathway in T-cells that are wildtype for BRAF, while the immune response mediated by PLX3397 is explained by the depletion of an immunosuppressive environment. Therefore, the major beneficial effects of combining PLX3397 and PLX4032 in SM1 are derived from increasing the infiltration of functionally activated T-cells into the tumor, in addition to the direct antitumor effect of PLX4032 on the *BRAF*^*V600E*^ mutant tumor.

Myeloid and other cells of the tumor microenviroment can produce factors that confer resistance to targeted therapies. This is of particular importance when using BRAF inhibitors, since it has been shown that stromal cells secreting HGF or TNF-α can reactivate the MAPK and PI(3)K-AKT signaling pathways to cause resistance to RAF inhibition [27-29]. However, in our studies we could not readily demonstrate any protective effects of myeloid cell co-culture on SM1 cells with regards to sensitivity to PLX4032. We also directly tested the protective effects of growth factor ligands by treating SM1 cells exposed to PLX4032 with cytokines. However, these cytokines did not decrease the SM1 cell sensitivity to BRAF inhibitors. Thus, the previously observed growth factor-mediated effect on PLX4032 sensitivity may be tumor model dependent.

SM1 is a cell line that is relatively resistant to PLX4032 (IC_50_ ≈ 15 μM) *in vitro* and accordingly it forms tumors that are hard to eradicate in *in vivo* mouse models. Although there is a rapid antitumor response with PLX4032, tumors nonetheless progress over time and mice have to be sacrificed within two to three weeks. The relative PLX4032 resistance of SM1 cells may be tied to the multiple genomic alterations present in these cells, such as deletion of *CDKN2A* and amplification of *BRAF*^*V600E*^ and *MITF* [12]. Hyper-activation of *BRAF*^*V600E*^ has been shown to be a mechanism of resistance to BRAF inhibitors [38]. As additional murine melanoma *BRAF*^*V600E*^ cell lines and corresponding mouse models of melanoma are developed, the effects of PLX4032 combined with other therapeutic agents such as CSF-1R inhibitors may lead to stronger synergistic antitumor responses.

## Conclusions

Combination therapy with the CSF-1R inhibitor PLX3397 and the oncogenic BRAF inhibitor vemurafenib result in superior antitumor effects compared to single agent treatment in a murine model of melanoma. The antitumor activity is mediated by both i) the inhibition of the immunosuppressive tumor microenvironment which increases intratumoral lymphocyte infiltration, and ii) enhanced functionality of the infiltrating lymphocytes. This data provides strong rationale for the continued clinical testing of PLX3397 with vemurafenib in patients with melanoma (as in trial NCT01826448).

## Additional file

Additional file 1: Table S1. Top up-regulated genes in macrophages in combined treatment group with significant GO terms. **Table S2.** Top down-regulated genes in macrophages in combined treatment group with significant GO terms. **Figure S1.** Polarization of macrophages and expression of IFN-γ mediated by PLX3397 and PLX4032.

## Abbreviations

ACT: Adoptive cell transfer
CSF-1: Colony stimulating factor-1
CSF-1R: Colony stimulating factor-1 receptor
DMAP: Differentiation map portal
HGF: Hepatocyte growth factor
MDSC: Myeloid derived suppressor cells
RRHO: rank-rank hypergeometric Overlap
RTK: Receptor tyrosine kinase
TIL: Tumor-infiltrating lymphocytes
TIM: Tumor-infiltrating myeloid cells
TNF-α: Tumor necrosis factor-α

## References

[1] Chapman PB, Hauschild A, Robert C, Haanen JB, Ascierto P, Larkin J (2011). Improved survival with vemurafenib in melanoma with BRAF V600E mutation. N Engl J Med.

[2] Flaherty KT, Puzanov I, Kim KB, Ribas A, McArthur GA, Sosman JA (2010). Inhibition of mutated, activated BRAF in metastatic melanoma. N Engl J Med.

[3] Sosman JA, Kim KB, Schuchter L, Gonzalez R, Pavlick AC, Weber JS (2012). Survival in BRAF V600-mutant advanced melanoma treated with vemurafenib. N Engl J Med.

[4] Flaherty KT, Robert C, Hersey P, Nathan P, Garbe C, Milhem M (2012). Improved survival with MEK inhibition in BRAF-mutated melanoma. N Engl J Med.

[5] Gabrilovich DI, Nagaraj S (2009). Myeloid-derived suppressor cells as regulators of the immune system. Nat Rev Immunol.

[6] Schreiber RD, Old LJ, Smyth MJ (2011). Cancer immunoediting: integrating immunity's roles in cancer suppression and promotion. Science.

[7] Kerkar SP, Restifo NP (2012). Cellular constituents of immune escape within the tumor microenvironment. Canc Res.

[8] Priceman SJ, Sung JL, Shaposhnik Z, Burton JB, Torres-Collado AX, Moughon DL (2010). Targeting distinct tumor-infiltrating myeloid cells by inhibiting CSF-1 receptor: combating tumor evasion of antiangiogenic therapy. Blood.

[9] Tarhini AA, Butterfield LH, Shuai Y, Gooding WE, Kalinski P, Kirkwood JM (2012). Differing patterns of circulating regulatory T cells and myeloid-derived suppressor cells in metastatic melanoma patients receiving anti-CTLA4 antibody and interferon-alpha or TLR-9 agonist and GM-CSF with peptide vaccination. J Immunother.

[10] Dai XM, Ryan GR, Hapel AJ, Dominguez MG, Russell RG, Kapp S (2002). Targeted disruption of the mouse colony-stimulating factor 1 receptor gene results in osteopetrosis, mononuclear phagocyte deficiency, increased primitive progenitor cell frequencies, and reproductive defects. Blood.

[11] Li J, Chen K, Zhu L, Pollard JW (2006). Conditional deletion of the colony stimulating factor-1 receptor (c-fms proto-oncogene) in mice. Genesis.

[12] Koya RC, Mok S, Otte N, Blacketor KJ, Comin-Anduix B, Tumeh PC (2012). BRAF Inhibitor Vemurafenib Improves the Antitumor Activity of Adoptive Cell Immunotherapy. Canc Res.

[13] Knight DA, Ngiow SF, Li M, Parmenter T, Mok S, Cass A (2013). Host immunity contributes to the anti-melanoma activity of BRAF inhibitors. J Clin Invest.

[14] Mok S, Koya RC, Tsui C, Xu J, Robert L, Wu L (2014). Inhibition of CSF-1 Receptor Improves the Antitumor Efficacy of Adoptive Cell Transfer Immunotherapy. Canc Res.

[15] Koya RC, Mok S, Comin-Anduix B, Chodon T, Radu CG, Nishimura MI (2010). Kinetic phases of distribution and tumor targeting by T cell receptor engineered lymphocytes inducing robust antitumor responses. Proc Natl Acad Sci U S A.

[16] Olivas E, Chen BB, Walker WS (1995). Use of the Pannell-Milstein roller bottle apparatus to produce high concentrations of the CSF-1, the mouse macrophage growth factor. J Immunol Methods.

[17] Sondergaard JN, Nazarian R, Wang Q, Guo D, Hsueh T, Mok S (2010). Differential sensitivity of melanoma cell lines with BRAFV600E mutation to the specific Raf inhibitor PLX4032. J Transl Med.

[18] Gentleman RC, Carey VJ, Bates DM, Bolstad B, Dettling M, Dudoit S (2004). Bioconductor: open software development for computational biology and bioinformatics. Genome Biol.

[19] Gautier L, Cope L, Bolstad BM, Irizarry RA (2004). affy–analysis of Affymetrix GeneChip data at the probe level. Bioinformatics.

[20] Smyth GK, Gentleman R, Carey V, Dudoit S, Irizarry R, Huber W: Bioinformatics and Computational Biology Solutions using R and Bioconductor. Limma: linear models for microarray data 2005:397–420.

[21] Gaujoux R, Seoighe C (2010). A flexible R package for nonnegative matrix factorization. BMC Bioinformatics.

[22] Novershtern N, Subramanian A, Lawton LN, Mak RH, Haining WN, McConkey ME (2011). Densely interconnected transcriptional circuits control cell states in human hematopoiesis. Cell.

[23] Plaisier SB, Taschereau R, Wong JA, Graeber TG (2010). Rank-rank hypergeometric overlap: identification of statistically significant overlap between gene-expression signatures. Nucleic Acids Res.

[24] Boyle EI, Weng S, Gollub J, Jin H, Botstein D, Cherry JM (2004). GO::TermFinder–open source software for accessing Gene Ontology information and finding significantly enriched Gene Ontology terms associated with a list of genes. Bioinformatics.

[25] Schilling B, Paschen A (2013). Immunological consequences of selective BRAF inhibitors in malignant melanoma: Neutralization of myeloid-derived suppressor cells. Oncoimmunology.

[26] Schilling B, Sucker A, Griewank K, Zhao F, Weide B, Gorgens A (2013). Vemurafenib reverses immunosuppression by myeloid derived suppressor cells. Int J Cancer.

[27] Straussman R, Morikawa T, Shee K, Barzily-Rokni M, Qian ZR, Du J (2012). Tumour micro-environment elicits innate resistance to RAF inhibitors through HGF secretion. Nature.

[28] Wilson TR, Fridlyand J, Yan Y, Penuel E, Burton L, Chan E (2012). Widespread potential for growth-factor-driven resistance to anticancer kinase inhibitors. Nature.

[29] Gray-Schopfer VC, Karasarides M, Hayward R, Marais R (2007). Tumor necrosis factor-alpha blocks apoptosis in melanoma cells when BRAF signaling is inhibited. Canc Res.

[30] Pyonteck SM, Akkari L, Schuhmacher AJ, Bowman RL, Sevenich L, Quail DF (2013). CSF-1R inhibition alters macrophage polarization and blocks glioma progression. Nat Med.

[31] Solinas G, Schiarea S, Liguori M, Fabbri M, Pesce S, Zammataro L (2010). Tumor-conditioned macrophages secrete migration-stimulating factor: a new marker for M2-polarization, influencing tumor cell motility. J Immunol.

[32] Michelucci A, Cordes T, Ghelfi J, Pailot A, Reiling N, Goldmann O (2013). Immune-responsive gene 1 protein links metabolism to immunity by catalyzing itaconic acid production. Proc Natl Acad Sci U S A.

[33] Glimcher LH, Townsend MJ, Sullivan BM, Lord GM (2004). Recent developments in the transcriptional regulation of cytolytic effector cells. Nat Rev Immunol.

[34] Gattinoni L, Klebanoff CA, Restifo NP (2012). Paths to stemness: building the ultimate antitumour T cell. Nat Rev Cancer.

[35] Hu-Lieskovan S, Robert L, Homet Moreno B, Ribas A. Combining Targeted Therapy With Immunotherapy in BRAF-Mutant Melanoma: Promise and Challenges. J Clin Oncol. 2014;32(21):2248–2254. 10.1200/JCO.2013.52.1377 PMC4164812 24958825

[36] Heidorn SJ, Milagre C, Whittaker S, Nourry A, Niculescu-Duvas I, Dhomen N (2010). Kinase-dead BRAF and oncogenic RAS cooperate to drive tumor progression through CRAF. Cell.

[37] Poulikakos PI, Zhang C, Bollag G, Shokat KM, Rosen N (2010). RAF inhibitors transactivate RAF dimers and ERK signalling in cells with wild-type BRAF. Nature.

[38] Shi H, Moriceau G, Kong X, Lee MK, Lee H, Koya RC (2012). Melanoma whole-exome sequencing identifies (V600E)B-RAF amplification-mediated acquired B-RAF inhibitor resistance. Nat Commun.

