# Supplementary material for: Inhibition of colony stimulating factor-1 receptor improves antitumor efficacy of BRAF inhibition
Source: BMC Cancer. 2015 May 5;15:356. doi: 10.1186/s12885-015-1377-8 (PMC4432503; doi:10.1186/s12885-015-1377-8)

Supplemental Table 1. Top up-regulated genes in macrophages in combined treatment group with significant GO terms

GOID	GO Term	P-value	Bonferroni P-value
GO:0034097	response to cytokine	4.82E-04	1.01E-06
GO:0035458	cellular response to interferon-beta	1.66E-03	3.47E-06
GO:0071345	cellular response to cytokine stimulus	2.26E-03	4.71E-06
GO:0035456	response to interferon-beta	3.06E-03	6.39E-06

Supplemental Table 2. Top down-regulated genes in macrophages in combined treatment group with significant GO terms

GOID	GO Term	P-value	Bonferroni P-value
GO:0001944	vasculature development	2.55E-11	1.62E-14
GO:0001568	blood vessel development	3.99E-11	2.54E-14
GO:0016477	cell migration	2.53E-10	1.61E-13
GO:0072358	cardiovascular system development	7.81E-10	4.97E-13
GO:0072359	circulatory system development	7.81E-10	4.97E-13
GO:0048870	cell motility	2.99E-09	1.90E-12
GO:0051674	localization of cell	2.99E-09	1.90E-12
GO:0048514	blood vessel morphogenesis	3.36E-08	2.14E-11
GO:0009611	response to wounding	4.44E-08	2.82E-11
GO:0040011	locomotion	1.10E-07	7.01E-11
GO:0001525	angiogenesis	1.80E-07	1.14E-10

Supplemental Figure Legends

Supplemental Figure 1. Polarization of macrophages and expression of IFN-γ mediated by PLX3397 and PLX4032. Heat maps for the top **a)** up-regulated and **b)** down-regulated genes in F4/80(+) CD11b(+) macrophages in combined treatment group. Color scale, log_2-transformed fold change expression (red, high; green, low) for each gene (row) normalized by the mean of all samples. **c)** Relative expression of IFN-γ mRNA in CD3(+) CD8(+) T-cells in SM1 tumors treated with PLX3397 and PLX4032 for 5 days. Biological replicates from each treatment group were shown. The level of IFN-γ expression reflected the activation status of T-cells. The IFN-γ expression in vehicle group was randomly scattered suggesting the T-cells were not well activated. All three drug-treatment groups had significant induction of IFN-γ expression.

Supplemental Figure 1

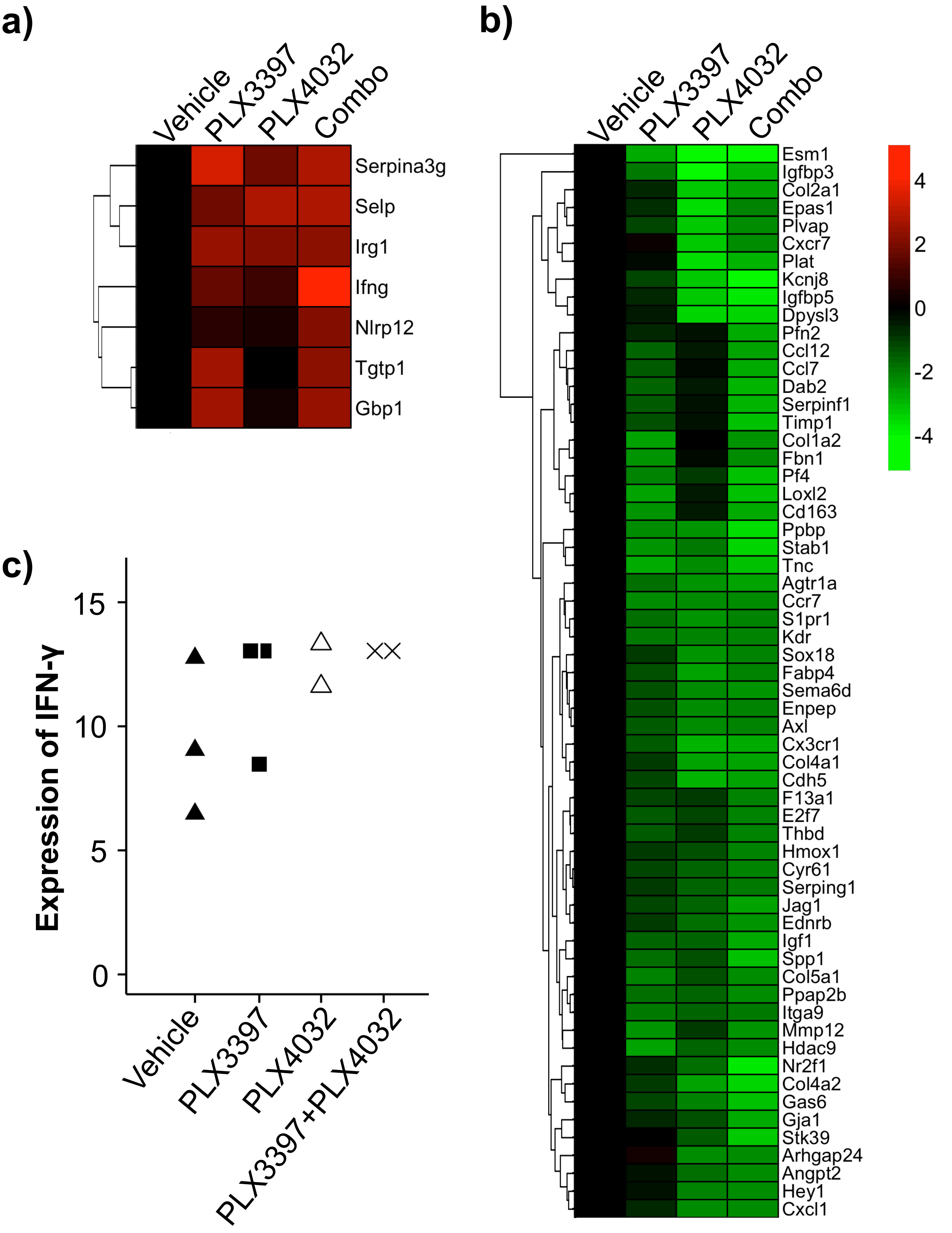

Supplement: Additional file 1: Table S1. — Top up-regulated genes in macrophages in combined treatment group with significant GO terms. Table S2. Top down-regulated genes in macrophages in combined treatment group with significant GO terms. Figure S1. Polarization of macrophages and expression of IFN-γ mediated by PLX3397 and PLX4032. [file 12885_2015_1377_MOESM1_ESM.pdf]
